# Research priorities in HIV, aging and rehabilitation: building on a framework with the Canada-International HIV and Rehabilitation Research Collaborative

**DOI:** 10.1186/s12981-023-00582-4

**Published:** 2023-12-09

**Authors:** Kelly K. O’Brien, Francisco Ibáñez-Carrasco, Kelly Birtwell, Graeme Donald, Darren A. Brown, Andrew D. Eaton, Bakita Kasadha, Emma Stanmore, Natalie St. Clair-Sullivan, Liam Townsend, Jaime H. Vera, Patricia Solomon

**Affiliations:** 1 Department of Physical Therapy, Temerty Faculty of Medicine, University of Toronto, 160-500 University Avenue, Toronto, ON M5G 1V7 Canada; 2 Institute of Health Policy, Management and Evaluation (IHPME), Dalla Lana School of Public Health, University of Toronto, 155 College Street, 4th Floor, Toronto, ON M5T 3M7 Canada; 3 Rehabilitation Sciences Institute (RSI), University of Toronto, 500 University Avenue, Room 160, Toronto, ON M5G 1V7 Canada; 4 Dalla Lana School of Public Health, University of Toronto, 155 College Street, 4th Floor, Toronto, ON M5T 3M7 Canada; 5 Centre for Primary Care and Health Services Research, University of Manchester, Oxford Road, Manchester, M13 9PL United Kingdom; 6 Division of Nursing, Midwifery and Social Work, Faculty of Biology, Medicine and Health, School of Health Sciences, University of Manchester, Oxford Road, Manchester, M13 9PL United Kingdom; 7 Therapies Department, Chelsea and Westminster Hospital NHS Foundation Trust, London, United Kingdom; 8 Faculty of Social Work, University of Regina, Saskatoon Campus, The Concourse, 111-116 Research Drive, Saskatoon, SK S7N 3R3 Canada; 9 Factor-Inwentash Faculty of Social Work, University of Toronto, 246 Bloor St W, Toronto, ON M5S 1V4 Canada; 10 Nuffield Department of Primary Care Health Sciences, Medical Sciences Division, University of Oxford, Radcliffe Primary Care Building, Radcliffe Observatory Quarter, Woodstock Road, Oxford, OX2 6GG United Kingdom; 11 Healthy Ageing Research Group (HARG), University of Manchester, Manchester, United Kingdom; 12 The Lawson Unit, Royal Sussex County Hospital, Brighton and Sussex University Hospitals NHS Trust, Eastern Road, Brighton, BN2 5BE United Kingdom; 13 Department of Infectious Diseases, St James’s Hospital, Dublin 8, Dublin, Ireland; 14 Department of Clinical Medicine, School of Medicine, Trinity Translational Medicine Institute, Trinity College Dublin, St. James’s Hospital, Dublin 8, Dublin, Ireland; 15 Department of Global Health and Infection, Brighton and Sussex Medical School, University of Sussex, Brighton, BN1 9PX East Sussex United Kingdom; 16 School of Rehabilitation Science, McMaster University, 1400 Main Street West, Room 403, Hamilton, ON L8S 1C7 Canada

**Keywords:** HIV, Disability, Rehabilitation, Research priorities, Aging

## Abstract

**Background:**

In 2016, the Canada-International HIV and Rehabilitation Research Collaborative established a framework of research priorities in HIV, aging and rehabilitation. Our aim was to review and identify any new emerging priorities from the perspectives of people living with HIV, clinicians, researchers, and representatives from community organizations.

**Methods:**

We conducted a multi-stakeholder international consultation with people living with HIV, researchers, clinicians and representatives of community-based organizations. Stakeholders convened for a one-day Forum in Manchester, United Kingdom (UK) to discuss research priorities via a web-based questionnaire and facilitated discussions. We analyzed data using conventional content analytical techniques and mapped emerging priorities onto the foundational framework.

**Results:**

Thirty-five stakeholders from the UK(n = 29), Canada(n = 5) and Ireland(n = 1) attended the Forum, representing persons living with HIV or representatives from community-based organizations(n = 12;34%), researchers or academics(n = 10;28%), service providers(n = 6;17%), clinicians(n = 4;11%); and trainees(n = 4;11%). Five priorities mapped onto the Framework of Research Priorities across three content areas: A–Episodic Health and Disability Aging with HIV (disability, frailty, social participation), B-Rehabilitation Interventions for Healthy Aging across the Lifespan (role, implementation and impact of digital and web-based rehabilitation interventions) and C–Outcome Measurement in HIV and Aging (digital and web-based rehabilitation health technology to measure physical activity). Stakeholders indicated methodological considerations for implementing digital and web-based rehabilitation interventions into research and practice and the importance of knowledge transfer and exchange among the broader community.

**Conclusion:**

Results highlight the sustained importance of the Framework of Research Priorities and provide further depth and areas of inquiry related to digital and web-based rehabilitation interventions and technology aging with HIV.

**Supplementary Information:**

The online version contains supplementary material available at 10.1186/s12981-023-00582-4.

## Introduction

People with HIV are living longer and aging with increased physical, mental and social health-related challenges [1]. COVID-19, alongside associated years of quarantine measures and shifts towards telemedicine, has increased health complexities and social isolation among adults living with HIV, highlighting the need for innovative interventions [2–4]. Rehabilitation interventions focused on physical and mental health have an important role in preventing and reducing disability for adults aging with HIV [5–7].

The World Health Organization defines ‘healthy aging’ as “the process of redeveloping and maintaining functional ability that enables well-being in older age”, recognizing the interaction between personal and environmental factors that influence health [8] Lazarus and colleagues (2016) proposed a fourth “90”, to the Joint United Nations Programme on HIV/AIDS (UNAIDS) global target of “90–90-90” stating that 90% of people living with HIV with undetectable viral load should report good health-related quality of life [9], which can be extrapolated to the updated 95-95-95 target [10]. Lazarus et al. (2023) went on to establish a Long Term Success Framework living with HIV, that highlighted optimizing health related quality of life as a key goal for persons living with HIV [11]. Achieving this success would require an integrated person-centered approach to care that goes beyond viral suppression to consider multimorbidity, and include comprehensive person-centred approaches to care that optimize self-perceived quality of life for persons living with HIV [9, 11–14]. Rehabilitation is well positioned to achieve long term success aging with HIV as it involves the dynamic process of prevention or treatment activities and services that address symptoms, functional limitations and social participation restrictions.

The Canada-International (originally Canada-UK) HIV and Rehabilitation Research Collaborative (CIHRRC) is a network of researchers, clinicians, people living with HIV, representatives from community organizations and policy stakeholders formed in 2009 with an aim to translate knowledge and identify emerging priorities in HIV and rehabilitation research [15]. In 2016, members of this collaborative convened in Canada to develop the Framework of Research Priorities in HIV, Aging and Rehabilitation comprised of seven research priorities: (i) nature, extent and impact of disability, concurrent health conditions and chronic inflammation with HIV; (ii) prevalence, severity and impact of frailty; (iii) community and social participation aging with HIV; (iv) strategies for chronic disease management and healthy aging with HIV; (v) facilitators and barriers to access and engagement in, rehabilitation; (vi) effectiveness of rehabilitation interventions for healthy aging with HIV; and (vii) advancing development and use of patient reported outcome measures in HIV and aging [16]. These seven priorities spanned three content areas: A–Episodic Health and Disability Aging with HIV; B-Rehabilitation Interventions for Healthy Aging across the Lifespan and C–Outcome Measurement in HIV and Aging. Since the development of this Framework, web-based interventions, digital health technology, and tele-health and rehabilitation models of care delivery have increasingly emerged in the context of HIV and aging [17–21]. As such, it is critical to revisit these research priorities seven years after the original development of the Framework, to consider their sustained relevance, identify new priorities, and establish a coordinated research response to address disability, and promote health outcomes among people aging with HIV.

Our aim was to review and build upon the previously published Framework of Research Priorities in HIV, Aging and Rehabilitation [16] to identify emerging research priorities from the perspectives of people living with HIV, clinicians, researchers, representatives from community organizations.

## Methods

We conducted a multi-stakeholder in-person international consultation with people living with HIV, researchers, clinicians and representatives of community-based organizations to identify priorities related to HIV, aging and rehabilitation. Stakeholders convened for a one-day in-person *International Forum on HIV, Aging and Rehabilitation Research: Aging in an Uncertain World,* held in collaboration with the Canada-International HIV and Rehabilitation Research Collaborative (CIHRRC), at the University of Manchester, UK on May 20, 2023. The objectives of the Forum were aimed at: (1) facilitating knowledge transfer and exchange on HIV and rehabilitation interventions (including online/tele-rehabilitation) to promote HIV and aging research and clinical practice; (2) establishing new research and clinical partnerships in HIV and aging internationally; (3) fostering mentorship and training in HIV and aging research; and (4) identifying emerging issues and research priorities in rehabilitation interventions for people aging with HIV. Our focus in this report is on the research priorities that emerged from this consultation in the context of HIV, aging and rehabilitation.

### Ethics

We reviewed the need for ethics approval with the University of Toronto Health Sciences Research Ethics Board and the University of Manchester Institutional Review Board who confirmed that given the consultative nature of the Forum, this work did not require ethics approval.

### Stakeholders

We invited people living with HIV, clinicians, academics, representatives from community-based organizations, community members, and members of the CIHRRC with interest and expertise in aging, HIV and rehabilitation. We promoted the Forum through email and website communications to members of CIHRRC, the University of Manchester, and community-based local organizations in Manchester, UK. Forum speakers further promoted the Forum via targeted invitations and word-of-mouth among stakeholders with an interest in HIV, aging and rehabilitation. 

### Forum program

Fourteen invited speakers from the UK, Canada and Ireland presented on research and models of community and clinical practice delivery among adults aging with HIV. The Forum included one keynote speaker, two research evidence sessions comprised of nine presentations, and one panel discussion with facilitated discussion throughout. The keynote presentation focused on an overview of *30 Years of HIV and Rehabilitation Research* and how the past may inform future research. The first research evidence session included five speaker presentations focused on the *Role of Mental Health in HIV and Aging and Rehabilitation*. The second research evidence session included four speaker presentations focused on *Frailty and Rehabilitation Interventions Aging with HIV*. The panel discussion session with five speakers focused on *Technology-Based Interventions for Enhancing Physical and Mental Health Outcomes for People Aging with HIV*. The Forum concluded with a large group discussion focused on identifying emerging issues, priority areas and next steps for future research. See Additional File 1 for the Forum Program. Videos and speaker slides of the Forum presentations are accessible here: https://cihrrc.ca/forums/2023-manchester-forum/.

Data pertaining to stakeholder perspectives on research priorities were collected using the following four strategies:

- Prior to the Forum, attendees were asked to submit responses to the following questions, ‘In your opinion, what have been key achievements in HIV, aging and rehabilitation research?’ and ‘In your opinion, what are 2–3 key research priorities in the area of HIV, aging and rehabilitation that are essential for moving the field forward?’ [strategy 1];
- During the Forum, two graduate trainee rapporteurs documented discussion during presentations and group discussion [strategy 2];
- During the Forum, attendees documented their ideas related to emerging research priorities on chart paper [strategy 3]; and
- After the Forum, attendees were asked to complete an evaluation form that included the following questions: ‘What are the three most important “take-home messages” that you heard at the Forum?’, and ‘In your opinion, what are 1 or 2 new and emerging issues related to HIV, aging and rehabilitation interventions that were not covered in the Forum?’ [strategy 4]

We used the collective responses, discussion, and feedback derived from these sources as the foundation for identifying the priorities. We collated and analyzed the data using conventional content analytical techniques [22]. We mapped the priorities onto the existing Framework of Research Priorities in HIV, Aging and Rehabilitation [16].

A core team (FIC, KKO, KB, GD) reviewed all sources of data, coded and clustered codes into categories to represent research priorities in HIV, aging and rehabilitation. These same members of the Core Team (KKO, FIC, KB, GD), met to review the data, identify research priority areas derived from the coding process, cluster the priority areas into broader content areas, and map them onto the original Framework of Research Priorities for HIV, Aging and Rehabilitation [16]. The priorities were circulated once to speakers and rapporteurs of the Forum for their review and refinement, and reviewed and finalized by members of the authorship team.

## Results

Of the 54 stakeholders who registered for the *International Forum on HIV, Aging and Rehabilitation Research* (19 speakers; 2 rapporteurs; 33 attendees), 35 (65%) attended the event from the UK (n = 29), Canada (n = 5) and Ireland (n = 1). The majority of stakeholders represented persons living with HIV or representatives from community-based organizations (n = 12; 34%) and/or researchers or academics (n = 10; 28%), service providers (n = 6; 17%), clinicians (n = 4; 11%); and trainees (n = 4; 11%). Researchers and clinicians were primarily rehabilitation professionals (physiotherapists or occupational therapists), physicians (infectious diseases), and nurses. Stakeholders worked in community-based organizations (n = 15; 43%), academic institutions (n = 12; 34%); hospital or community healthcare organizations (n = 6; 17%), or in non-governmental or private organization (n = 2; 6%). Of the 35 stakeholders, 19 (54%) were speakers at the Forum.

### Research priorities in HIV, aging and rehabilitation

Evidence presented at the Forum aligned with the Framework of Research Priorities in HIV, Aging and Rehabilitation by addressing the following areas: disability, frailty, social participation, access to and impact of rehabilitation, and patient-reported outcome measures in HIV and aging. The focus on rehabilitation interventions and strategies for healthy aging with HIV included digital health and web-based interventions and supportive networks.

Five priorities emerged from the Forum, all of which aligned with the original priorities, and three component content areas of the original 2016 Framework [16]: A–Episodic Health, Multimorbidity and Disability Aging with HIV (i) episodic disability and uncertainty, ii) frailty, iii) social participation); B–Rehabilitation Interventions for Healthy Aging across the Lifespan (iv) examining the role, implementation and impact of digital and web-based rehabilitation interventions with adults aging with HIV) and C–Outcome Measurement in HIV and Aging (v) using digital health technology to measure physical activity). Stakeholders highlighted the importance of learning from the broader aging and rehabilitation field, and the importance of knowledge transfer and exchange among researchers, clinicians, people living with HIV, trainees, and the broader community (Table 1). The research priorities related to web-based and digital health interventions build on the priorities in the original Framework, strengthening the scaffold for collaborations on research related to rehabilitation interventions to promote healthy aging with HIV. We describe more detail in each of the priorities areas below.

**Table 1:** Research Priorities in HIV, Aging and Rehabilitation: Building on a Framework with the Canada-International HIV and Rehabilitation Research Collaborative

Component Priority Content Area	Research priorities in 2016 (O’Brien et al. 2020) [16]	Research priorities in 2023	Methodological Considerations
A – Episodic health, multi-morbidity and disability aging with HIV	**Priority 1—**Nature, extent and impact of disability, concurrent health conditions and chronic inflammation with HIV	(i) Episodic disability & uncertainty	• Stigma-reducing strategies to facilitate engagement of adults aging with HIV • Culturally safe, anti-oppressive and age-sensitive approaches to research on rehabilitation to better engage adults aging with HIV • Community-engaged approaches involving persons living with HIV at all stages of the research • Implementation science approaches to examine rehabilitation interventions in ‘real-world’ settings • Person-centred approaches; not a one-size-fits all approach; • Digital and web-based rehabilitation technologies: consider roles for old and newer technologies in rehabilitation and as a catalyst to facilitate clear communication
	**Priority 2—**Prevalence, severity and impact of frailty	(ii) Frailty & other concurrent health conditions	
	**Priority 3**—Community and social participation aging with HIV	(iii) Social participation, engagement and relationships among adults aging with HIV	
B-Rehabilitation interventions for healthy aging across the lifespan	**Priority 4—**Strategies for chronic disease management and healthy aging with HIV	iv) Role, implementation and impact of digital and web-based rehabilitation interventions and health technologies (uptake and usage, examining person-centred approaches, models of implementation of web-based and digital health and rehabilitation technology, cost, accessibility)	
	**Priority 5**—Examining facilitators and barriers to access and engagement in rehabilitation		
	**Priority 6—**Determining the effectiveness of rehabilitation interventions to support healthy aging with HIV (including wireless physical activity monitors, online apps, websites, social media, online tele-coaching)		
C-Outcome measurement in HIV and aging research	**Priority 7**—Advancing development and use of patient reported outcome measures in HIV, aging, and rehabilitation	(v) Using digital and web-based rehabilitation health technology to measure engagement in physical activity	
**Knowledge translation and exchange** Importance of mobilizing research into practice, programs and policy with researchers, clinicians, students, people living with HIV and the broader HIV community to enhance timely, and appropriate rehabilitation for persons aging with HIV			

#### Component A – Episodic health, multimorbidity and disability aging with HIV

Stakeholders emphasized the importance of addressing i) episodic disability and uncertainty, ii) frailty, and iii) social participation among adults aging with HIV across the lifespan (which align with Priorities 1–3 in the original Framework) (Table 1).

i. *Episodic disability & uncertainty*
  Stakeholders highlighted the importance of examining the nature and severity of episodic disability (physical, cognitive, mental-emotional, daily activities, social inclusion), and specifically the uncertainty that may be experienced among adults aging with HIV across the lifespan. This included examining episodic disability in the context of longstanding and historic HIV pharmacological interventions among older adults with HIV.
ii. *Frailty and other concurrent health conditions*
  Stakeholders identified the importance of examining disability associated with frailty in combination with other health conditions across the life span, including but not limited to, osteoporosis, menopause, chronic pain, cardiovascular disease. Considerations of biological age versus time since HIV diagnosis was highlighted by stakeholders and the importance of considering the life course of adults aging with HIV (regardless of time of HIV diagnosis) and distinguishing between different phenotypic forms of frailty: weakness, slowness, exhaustion, low physical activity, and unintentional weight loss. Stakeholders highlighted an evidence to practice gap on frailty, aging and HIV and the need for a better understanding among researchers and clinicians on how to prevent, detect and address frailty in adults aging with HIV.
iii. *Social participation, engagement and relationships among adults aging with HIV*
  Stakeholders highlighted the importance for researchers to consider the social and interpersonal needs of adults aging with HIV and the emotional, sexual health, intimacy and connectedness associated with relationships among adults aging with HIV. While stakeholders acknowledged this priority in the original Framework, they emphasized the relevance of social connectedness due to a lack of in-person interactions during the COVID-19 pandemic. Stakeholders discussed how the COVID-19 pandemic necessitated changes in how people interacted socially and maximized the use of digital resources where possible. While stakeholders acknowledged positively enhancements to social interactions with digital technology, others cautioned the digital divide, lack of access and digital literacy that could limit social engagement among adults aging with HIV.

#### Component B—Rehabilitation interventions for healthy aging across the lifespan

Stakeholders highlighted the need to examine the role, implementation and impact of digital health and web-based rehabilitation technologies, which aligned with Priority 6 in the original Framework. This examines the role of technology (wireless physical activity monitors, online apps, websites, social media, online tele-coaching) in augmenting engagement in rehabilitation interventions among adults aging with HIV (Table 1).

iv. *Role, implementation and impact of digital and web-based rehabilitation interventions and health technologies*
  Stakeholders discussed the need to examine the uptake and usage of digital health technologies among adults aging with HIV and health and rehabilitation providers, and evidence-based person-centred approaches to digital technology and web-based rehabilitation interventions. Stakeholders also raised the potential role and impact of artificial intelligence for rehabilitation (adaptation, implementation, trust) among adults aging with HIV. Stakeholders highlighted the importance of considering the costs associated with implementation of digital health interventions, and financial barriers to accessing online interventions, which may be limited by financial, policy, or programmatic barriers. Future research should examine different models of implementation of digital health technology, their cost and accessibility in the context of aging with HIV.

#### Component C – Outcome measurement in HIV and aging research

Stakeholders identified the potential role for digital health technology to measure engagement in physical activity among adults aging with HIV, which aligned with Priority 7 in the original Framework (advancing the development and use of patients reported outcome measures in HIV, aging and rehabilitation) (Table 1).

v. * Using digital and web-based rehabilitation health technology to measure physical activity*
  Stakeholders identified the potential for evaluating the impact of rehabilitation interventions including web-based interventions and digital health tools (such as wearables, wireless physical activity monitors, apps, gamification) on engagement in social connectedness interventions, and physical activity and the potential impact on disability and health outcomes among adults aging with HIV.
  Collectively, stakeholders acknowledged that digital health technology in HIV and aging was not a one-size fits all approach, nor a replacement for in-person health and rehabilitation services or HIV care. There still exists a role and importance and need for in-person models of delivery, and while basic forms of technology (e.g. telephone for fostering peer-support) may be simple, they can be effective. While technology may serve as a potential tool to facilitate engagement and mitigate fragmentation of health, social and rehabilitation services for persons aging with HIV, there is a need to balance technology interventions with individual need, access, literacy and comfort.

#### Methodological considerations, knowledge translation and exchange

Methodological considerations for addressing these research priorities also emerged from the consultation that complemented the original *Framework of Research Priorities*. Stakeholders recommended that researchers consider barriers to engaging in research, such as stigma, and the need for culturally safe, anti-oppressive and age-sensitive approaches to research on rehabilitation to better engage adults aging with HIV. For example, strategies for better engaging women, Indigenous and Métis adults aging with HIV in colonized countries such as Canada, persons who use drugs, racialized groups, individuals in rural geographical regions as well as those who may be experiencing stigma and fear of disclosure. Community-engaged approaches, involving people living with HIV in all aspects of the rehabilitation research is critical for ensuring the research is meaningful and relevant to the community.

Given the focus on rehabilitation interventions, attendees highlighted the importance of implementation science approaches for assessing how interventions are taken up and their impact in the ‘real world’ setting, and how interventions might be adopted in the broader context of policy and programs for adults aging with HIV [23, 24]. Attendees highlighted the need to examine public health policies and the importance of linking research to practice and programs that improve health outcomes for adults aging with HIV. Specific methodological considerations raised by stakeholders pertained to digital health technology, including the importance of implementing digital and web-based rehabilitation interventions into research and practice. Technology was highlighted as a potential catalyst or channel to facilitate communication among patients and providers (not the end goal or intervention itself).

Some final considerations, recommendations or key messages from the Forum included: recognizing that old and new technologies are both important for enhancing rehabilitation among adults aging with HIV, including engaging in physical activity, and fostering personal connections with other peers, health or rehabilitation providers, or fitness personnel. Stakeholders described technology as a vehicle of communication, and the importance of clear communication to wide audiences pertaining to terms in areas of physical rehabilitation and aging such as frailty, vulnerability, susceptibility and social vulnerability. Of note, the concept, and the very mention, of aging can yield different conceptualizations for different people and is important to consider in approaching research.

## Discussion

The five priorities raised by stakeholders align with and build upon the original *Framework of Research Priorities in HIV, Aging and Rehabilitation* in all areas of disability, rehabilitation interventions, and outcomes focusing on the role, implementation and impact of digital health technology in HIV, aging and rehabilitation. Stakeholders outlined the overlapping importance of examining episodic disability in the context of rehabilitation interventions; and the use of digital health technology dually as an intervention and outcome measure of physical activity.

Digital health technology emerged from the Forum as a potential mechanism for rehabilitation, HIV assessment and interventions among adults aging with HIV. This was a reflection of the Forum program, which comprised of research evidence presentations on online forms of rehabilitation and HIV care delivery. The COVID-19 pandemic changed the course of health care delivery and thrust online models of HIV and rehabilitation care delivery into the forefront of care (3, 25). The COVID-19 pandemic contributed to the complexity of health challenges (e.g. social isolation, mental health) and disrupted models of rehabilitation care delivery. Technology has been used widely for education, accessing health and rehabilitation services, and implementing interventions in the context of HIV; this includes smartphone and app technology to facilitate engagement in care, medication adherence, neurocognitive assessment, and exercise [26–29], virtual driving test platforms to assess the ability to drive among persons with HIV and neurocognitive impairment [30], and tele-health exercise interventions with adults aging with HIV [20, 31, 32]. Artificial intelligence also is used with older adults for remote patient monitoring and smart home technology [33–35], assessment of mobility with mobility disorders [36, 37] or dementia [38], as well as facilitating rehabilitation assessment and treatment in order adults [39]. Nevertheless, stakeholders in this consultation highlighted that online forms of rehabilitation were not a one-size-fits-all, and not always a replacement for in-person interventions. This was supported by evidence reporting variable uptake and declining use of wireless physical activity monitors (WPAMs) among adults living with HIV engaged in a community-based exercise intervention [21] and barriers and facilitators to uptake of WPAMs in the context of HIV [40]. Similarly, the priorities from this Forum are supported by variability in digital health literacy documented among older adult populations during the COVID-19 pandemic [41, 42]. Stakeholders also highlighted the limitations of technology, balancing online and in-person approaches, and taking individualized approaches for weaving digital health technology into rehabilitation HIV care. They identified the need for evidence supporting the cost-effectiveness and sustainability of online rehabilitation interventions. Considerations implementing rehabilitation interventions using online or digital forms of technology should consider person-specific approaches taking into account accessibility, digital literacy, and delivery in the context of HIV [43].

The role, implementation and impact of digital health technology was closely interwoven with the priority of examining social connectedness and participation among adults aging with HIV. This was not surprising given the impact of COVID-19 pandemic on increases in disability, specifically uncertainty and mental health and social connectedness among persons living with HIV [4]. Furthermore, adults aging with HIV can face barriers of stigma, lack of education or a supportive or supervised environment to engage in rehabilitation interventions such as physical activity or exercise [44]. Rehabilitation interventions focused on enhancing social connections online is a potential intervention but may be difficult for some, and may not occur naturally in online settings. Future work should consider online rehabilitation interventions that allocate dedicated time for users to interact with each other and establish a comfort and rapport in an online setting [43].

The original *Framework of Research Priorities for HIV, Aging and Rehabilitation* [16] still resonated with stakeholders who suggested important updates, refinements and emerging issues. While encouraging to see that research in this field will continue to align with community need, it is also disheartening to see how little progress is felt by people living with HIV and other stakeholders regarding these priorities. This may be attributed to the pace and extent to which HIV and rehabilitation research is funded, implemented, and translated to practice. Furthermore, while these priorities appear to recognize the complexity of mental health and social connectedness faced by adults aging with HIV following the COVID-19 pandemic, this work highlights the ongoing relevance and importance of these priorities given the increasing proportion of older adults aging with HIV [4, 45]. Furthermore, these priorities align with, and complement the Long Term Success Framework by Lazarus and colleagues (2023) which outlines a holistic approach to the care of people aging with HIV spanning sustained undetectable viral load, minimal impact of treatment and clinical monitoring, optimizing health-related quality of life, lifelong integration of care, and freedom of discrimination [11]. Within the health-related quality of life pillar, authors highlight the role of patient-reported outcome measures in identifying and addressing needs of persons aging with HIV, empowering them to actively engage in their healthcare plan, which specifically aligns with Component C -Outcome Measurement in HIV and Aging Research in our Framework. Overall, results provide a focus on priorities related to disability, and digital health technology and highlight ongoing issues of implementation, methodological considerations and knowledge translation in the field for those aging with HIV.

These research priorities were developed from the perspectives of a multidisciplinary group of stakeholders with longstanding clinical, research and lived experiential expertise in HIV, aging and rehabilitation. We used a community-engaged approach involving people living with HIV in the consultation and development of the priorities. We built on the foundational *Framework of Research Priorities in HIV, Aging and Rehabilitation* [16] which subsequently was built on preceding research priorities [46], ensuring we identified current pertinent issues. Our team has strong history of research practice and lived expertise in HIV rehabilitation and aging.

## Limitations

This study has numerous limitations. We did not use a formal Delphi or nominal group technique to identify the priorities. Nevertheless we used multiple strategies to elicit perspectives on priorities such as web-based and in-person discussions. Our consultation was focused to stakeholders within Canada and the UK, and lacked representation of stakeholders from other high-income countries as well as low to middle-income countries conducting work in HIV, aging and disability. While these priorities were developed through a rehabilitation lens, addressing them will require collaborative and interprofessional and community-engaged approaches involving HIV, primary and geriatric care teams, social work, and psychology, in addition to rehabilitation to move the field forward. We acknowledge the field is continually evolving and new priorities will emerge as the course of HIV progresses and the role for rehabilitation in the context of HIV continues to grow.

## Conclusions

Results from the stakeholder consultation indicated the sustained relevance and importance of the original *Framework of Research Priorities in HIV, Aging and Rehabilitation* [16], while highlighting further depth and areas of inquiry related to digital and web-based rehabilitation interventions and technology in the context of aging with HIV. Findings offer a foundation for collaboration in future research and practice. Specific considerations to foster timely, appropriate and effective rehabilitation involving web-based digital rehabilitation interventions and models of delivery are needed.

### Supplementary Information

**Additional file 1: **Forum Program at a Glance—5th International Forum on HIV and Rehabilitation Research: Aging with HIV in an Uncertain World.

## Data Availability

The data supporting the conclusions of this article are included within the report. The datasets used and/or analyzed for this brief report are available from the corresponding author on reasonable request.

## Abbreviations

AI: Artificial intelligence
CIHRRC: Canada-International HIV and Rehabilitation Research Collaborative
UNAIDS: United Nations programme on HIV/AIDS
WPAM: Wireless physical activity monitor

## References

[1] The Lancet Healthy (2022). Ageing with HIV. Lancet Healthy Longev.

[2] Gatechompol S, Avihingsanon A, Putcharoen O, Ruxrungtham K, Kuritzkes DR (2021). COVID-19 and HIV infection co-pandemics and their impact: a review of the literature. AIDS Res Ther.

[3] SeyedAlinaghi S, Mirzapour P, Pashaei Z, Afzalian A, Tantuoyir MM, Salmani R (2023). The impacts of COVID-19 pandemic on service delivery and treatment outcomes in people living with HIV: a systematic review. AIDS Res Ther.

[4] O'Brien KK, Bayoumi AM, Chan Carusone S, Davis AM, Aubry R, Avery L (2021). Disability and self-care living strategies among adults living with HIV during the COVID-19 pandemic. AIDS Res Ther.

[5] deBoer H, Andrews M, Cudd S, Leung E, Petrie A, Chan Carusone S (2019). Where and how does physical therapy fit? Integrating physical therapy into interprofessional HIV care. Disabil Rehabil.

[6] Quigley A, O'Brien K, Parker R, MacKay-Lyons M (2019). Exercise and cognitive function in people living with HIV: a scoping review. Disabil Rehabil.

[7] Solomon P, Salbach NM, O'Brien KK, Nixon S, Baxter L, Gervais N (2019). Evaluation of a community-based self-management program to increase access to rehabilitation for people living with HIV. J Int Assoc Providers AIDS Care.

[8] Michel JP, Sadana R (2017). "Healthy aging" concepts and measures. J Am Med Dir Assoc.

[9] Lazarus JV, Safreed-Harmon K, Barton SE, Costagliola D, Dedes N, Del Amo VJ (2016). Beyond viral suppression of HIV—the new quality of life frontier. BMC Med.

[10] Frescura L, Godfrey-Faussett P, Feizzadeh AA, El-Sadr W, Syarif O, Ghys PD (2022). Achieving the 95 95 95 targets for all: a pathway to ending AIDS. PLoS ONE.

[11] Lazarus JV, Wohl DA, Cascio M, Guaraldi G, Rockstroh J, Hodson M (2023). Long-term success for people living with HIV: a framework to guide practice. HIV Med.

[12] Lazarus JV, Safreed-Harmon K, Kamarulzaman A, Anderson J, Leite RB, Behrens G (2021). Consensus statement on the role of health systems in advancing the long-term well-being of people living with HIV. Nat Commun.

[13] Lazarus JV, Cascio M, Anderson J, Pasanen S, Harding R (2023). A person-centred approach to enhance the long-term health and wellbeing of people living with HIV in Europe. J Int AIDS Soc.

[14] Fuster-RuizdeApodaca MJ, Wohl DA, Cascio M, Guaraldi G, Rockstroh J, Hodson M (2023). Why we need to re-define long-term success for people living with HIV. HIV Med.

[15] O’Brien KK, Solomon P, Ibáñez-Carrasco F, Chegwidden W, McDonnell E, Brown D (2018). Evolution of an international research collaborative in HIV and rehabilitation: community engaged process, lessons learned, and recommendations. Progress Commun Health Partnerships Res Educ Action.

[16] O'Brien KK, Ibanez-Carrasco F, Solomon P, Harding R, Brown D, Ahluwalia P (2020). Research priorities for rehabilitation and aging with HIV: a framework from the Canada-International HIV and rehabilitation research collaborative (CIHRRC). AIDS Res Ther.

[17] Noar SM (2011). Computer technology-based interventions in HIV prevention: state of the evidence and future directions for research. AIDS Care.

[18] Horvath KJ, Danilenko GP, Williams ML, Simoni J, Amico KR, Oakes JM (2012). Technology use and reasons to participate in social networking health websites among people living with HIV in the US. AIDS Behav.

[19] Schnall R, Cho H, Mangone A, Pichon A, Jia H (2018). Mobile health technology for improving symptom management in low income persons living with HIV. AIDS Behav.

[20] O'Brien KK, Ibanez-Carrasco F, Carusone SC, Bayoumi AM, Tang A, McDuff K (2023). Piloting an online telecoaching community-based exercise intervention with adults living with HIV: protocol for a mixed-methods implementation science study. BMJ Open.

[21] Turner JR, Chow J, Cheng J, Hassanali F, Sevigny H, Sperduti M (2023). Wireless physical activity monitor use among adults living with HIV in a community-based exercise intervention study: a quantitative, longitudinal, observational study. BMJ Open.

[22] Hsieh HF, Shannon SE (2005). Three approaches to qualitative content analysis. Qual Health Res.

[23] Greenhalgh T, Abimbola S (2019). The NASSS framework—a synthesis of multiple theories of technology implementation. Stud Health Technol Inform.

[24] Glasgow RE, Eckstein ET, Elzarrad MK (2013). Implementation science perspectives and opportunities for HIV/AIDS research: integrating science, practice, and policy. J Acquir Immune Defic Syndr.

[25] Budak JZ, Scott JD, Dhanireddy S, Wood BR (2021). The impact of COVID-19 on HIV care provided via telemedicine-past, present, and future. Curr HIV/AIDS Rep.

[26] Aomori M, Matsumoto C, Takebayashi S, Matsuyama N, Uto Y, Tanaka M (2023). Effects of a smartphone app-based diet and physical activity program for men living with HIV who have dyslipidemia: a pilot randomized controlled trial. Jpn J Nurs Sci.

[27] Bonato M, Turrini F, Meloni VDEZ, Plebani A, Brambilla M (2020). A mobile application for exercise intervention in people living with HIV. Med Sci Sports Exerc..

[28] Hightow-Weidman L, Muessig K, Knudtson K, Srivatsa M, Lawrence E, LeGrand S (2018). A gamified smartphone app to support engagement in care and medication adherence for HIV-positive young men who have sex with men (AllyQuest): development and pilot study. JMIR Public Health Surveill.

[29] Robbins RN, Brown H, Ehlers A, Joska JA, Thomas KG, Burgess R (2014). A Smartphone app to screen for HIV-related neurocognitive impairment. J Mob Technol Med.

[30] Grethlein D, Pirrone V, Devlin KN, Dampier W, Szep Z, Winston FK (2022). Examining virtual driving test performance and its relationship to individuals with HIV-associated neurocognitive disorders. Front Neurosci.

[31] Oursler KK, Marconi VC, Briggs BC, Sorkin JD, Ryan AS (2022). Telehealth exercise intervention in older adults with HIV protocol of a multisite randomized trial. J Assoc Nurses AIDS Care.

[32] Jennings SC, Manning KM, Bettger JP, Hall KM, Pearson M, Mateas C (2020). Rapid transition to telehealth group exercise and functional assessments in response to COVID-19. Gerontol Geriatr Med.

[33] Vandenberk B, Raj SR (2023). Remote patient monitoring: what have we learned and where are we going?. Curr Cardiovasc Risk Rep.

[34] Forchuk C, Serrato J, Lizotte D, Mann R, Taylor G, Husni S (2022). Developing a smart home technology innovation for people with physical and mental health problems: considerations and recommendations. JMIR Mhealth Uhealth.

[35] Aggar C, Sorwar G, Seton C, Penman O, Ward A (2023). Smart home technology to support older people's quality of life: a longitudinal pilot study. Int J Older People Nurs.

[36] Nguyen H, Lebel K, Boissy P, Bogard S, Goubault E, Duval C (2017). Auto detection and segmentation of daily living activities during a Timed Up and go task in people with Parkinson's disease using multiple inertial sensors. J Neuroeng Rehabil.

[37] Duffy O, Synnott J, McNaney R, Brito Zambrano P, Kernohan WG (2021). Attitudes toward the use of voice-assisted technologies among people with parkinson disease: findings from a web-based survey. JMIR Rehabil Assist Technol.

[38] Arthanat S, Wilcox J, LaRoche D (2022). Smart home automation technology to support caring of individuals with Alzheimer's disease and related dementia: an early intervention framework. Disabil Rehabil Assistive Technol.

[39] Alsobhi M, Sachdev HS, Chevidikunnan MF, Basuodan R (2022). Facilitators and barriers of artificial intelligence applications in rehabilitation: a mixed-method approach. Int J Environ Res Publ Health.

[40] Dagenais M, Cheng D, Salbach NM, Brooks D, O'Brien KK (2018). Wireless physical activity monitor use among adults living with HIV: a scoping review. Rehabilitation Oncol.

[41] Finkelstein R, Wu Y, Brennan-Ing M (2023). Older adults' experiences with using information and communication technology and tech support services in New York City: findings and recommendations for post-pandemic digital pedagogy for older adults. Front Psychol.

[42] Marston HR, Ivan L, Rosenberg D, Ratzenboeck B (2023). Editorial: post-pandemic digital realities of older adults. Front Psychol.

[43] Lau B, Sharma I, Manku S, Kobylianski J, Wong LY, Ibanez-Carrasco F (2022). Considerations for developing and implementing an online community-based exercise intervention with adults living with HIV: a qualitative study. BMJ Open.

[44] Chetty L, Cobbing S, Chetty V (2022). The perceptions of older people living with HIV/aids towards physical activity and exercise. AIDS Res Ther.

[45] Stanton AM, Goodman GR, Blyler A, Kirakosian N, Labbe AK, Robbins GK (2023). Mental health, social connectedness, and fear during the COVID-19 pandemic: a qualitative perspective from older women with HIV. AIDS Behav.

[46] O'Brien KK, Ibanez-Carrasco F, Solomon P, Harding R, Cattaneo J, Chegwidden W (2014). Advancing research and practice in HIV and rehabilitation: a framework of research priorities in HIV, disability and rehabilitation. BMC Infect Dis.

