# Supplementary material for: Research priorities in HIV, aging and rehabilitation: building on a framework with the Canada-International HIV and Rehabilitation Research Collaborative
Source: AIDS Res Ther. 2023 Dec 9;20:86. doi: 10.1186/s12981-023-00582-4 (PMC10709904; doi:10.1186/s12981-023-00582-4)
Supplement: Supplementary file 1 — Additional file 1: Forum Program at a Glance—5th International Forum on HIV and Rehabilitation Research: Aging with HIV in an Uncertain World. [file 12981_2023_582_MOESM1_ESM.pdf]

5th International Forum on HIV and Rehabilitation Research
Ageing with HIV in an Uncertain World
Saturday May 20, 2023 - 9:00am – 5:00pm, UK time

University of Manchester (In-Person) – Jean McFarlane Building, Rm G306A&B

Link to Online Program: https://bit.ly/3zeDWZG

PROGRAMME AT A GLANCE

Aim of Forum: To advance knowledge transfer and exchange, collaborations and partnerships in rehabilitation in the context of HIV and rehabilitation research and practice and to identify new and emerging research priorities in rehabilitation-focused research in HIV and ageing.

Time	Topic
830-900am	**Registration (Coffee & Tea)**
900-915am	**Welcome and Introductions – Graeme Donald & Kelly Birtwell,** *University of Manchester*
915-930am	**Overview of the Forum – Kelly O'Brien & Francisco Ibanez-Carrasco,** *University of Toronto*<br>**Moderator – Darren Brown,** *Chelsea and Westminster Hospital NHS Foundation Trust*
930-1015am	**Keynote Speaker – Dr. Patty Solomon,** *Professor Emeritus, McMaster University, Canada*<br>**30 Years of HIV and Rehabilitation Research – Can the Past Inform the Future?**
1015-1030am	**Break**
1030am-1200pm	**Research Evidence Session #1: Role of Mental Health in HIV and Ageing and Rehabilitation**<br>• **Ageing with HIV: Psychosocial challenges and implications**<br>Dana Rosenfeld, *University of Westminster, UK*<br>• **Holistic support for an ageing cohort**<br>Anna Hughes, *George House Trust, Manchester, UK*<br>• **Delivering Mindfulness-Based Interventions for People Living with HIV**<br>Kelly Birtwell & Graeme Donald, *University of Manchester, Manchester UK*<br>• **Implementation of frailty screening in people living with HIV: lessons from the Silver Clinic**<br>Jaime Vera, *The Silver Clinic, Brighton and Sussex University Hospital NHS Foundation Trust*<br>• **Access to service for people living with HIV experiencing neurocognitive difficulties**<br>Francisco Ibanez-Carrasco, *University of Toronto, Canada*
1200-100pm	**Lunch (provided to all attendees)**
100-215pm	**Panel: Technology-Based Interventions for enhancing physical and mental health outcomes for people ageing with HIV –** This dynamic session includes Anita Binns (George House Trust), Anna Hughes (George House Trust), Jo Josh (British HIV Association), Emma Stanmore (University of Manchester), and Brittany Torres (University of Toronto).
215-230pm	**Break**
230-345pm	**Research Evidence Session #2: Frailty and Rehabilitation Interventions Ageing with HIV**<br>• **Exploring frailty and frailty screening for older people living with HIV**<br>Natalie St. Clair-Sullivan, *Brighton and Sussex Medical School, UK*<br>• **Investigating and modifying frailty in People Living with HIV**<br>Liam Townsend, *St. Vincent's University Hospital, Dublin, Ireland*<br>• **Cognitive remediation group therapy as a hybrid strategy for people aging with HIV to cope with cognitive health concerns**<br>Andrew Eaton, *University of Regina, Regina, Saskatchewan, Canada*<br>• **Piloting an Online Tele-Coaching Community-Based Exercise Intervention Study with Adults Living with HIV: An Implementation Science Approach**<br>Kelly O'Brien, *University of Toronto, Canada*
345-400pm	**Break**
400-430pm	**Identifying Emerging Issues, Priority Areas and Next Steps for Future Research**
430-500pm	**Wrap Up, Evaluation, Acknowledgements and Next Steps**

Acknowledgements: This Forum is funded by a University of Manchester-University of Toronto partnership grant. We also acknowledge support from the Canada-International HIV and Rehabilitation Research Collaborative (CIHRRC), Rehabilitation in HIV Association (RHIVA), and Gay Men's Health Collective (GMHC) (filming). The Forum is supported by the Department of Physical Therapy and Dalla Lana School of Public Health, University of Toronto and the Division of Nursing, Midwifery and Social Work, University of Manchester.

Date Last Revised: May 15, 2023
